# Formalization of a new stock trend prediction methodology based on the sector price book value for the Colombian market

**DOI:** 10.1016/j.heliyon.2022.e09210

**Published:** 2022-03-31

**Authors:** Monroy-Perdomo Leonardo, Cardozo-Munar Carlos Eduardo, Torres-Hernández Ana María, Tena-Galeano José Luis, López-Rodríguez Campo Elias

**Affiliations:** a Corporación Universitaria Minuto de Dios - UNIMINUTO, IDEAS and ECONOMÍA SOLIDARIA Y DESARROLLO Research Groups, Colombia; b GECOEMPRESARIAL Research Group, Financial Administration Program of the Corporación Universitaria Minuto de Dios - UNIMINUTO, Colombia; c Escuela de Economía Solidaria of the Corporación Universitaria Minuto de Dios - UNIMINUTO, Research Group Economía Solidaria y Desarrollo (Solidarity Economy and Development), Colombia; d Business Administration Program of the Corporación Universitaria Minuto de Dios - UNIMINUTO, IDEAS Research Groups, Colombia; e Specialization in Financial Management Program of Corporacion Universitaria Minuto de Dios - UNIMINUTO, Colombia

**Keywords:** Capital markets, Tobin's Q, Financial markets, Corporate valuation, Trend analysis, Innovation, Competitiveness

## Abstract

As a financial indicator, Tobin's Q is related to making investment decisions based on the organization's market value related to the organization's replacement costs. The purpose of this study is to develop a methodology for predicting the trend of stock value based on the relationship between the fluctuation of Tobin's Q by sector as a trend index and the stock price variation of the Columbia stock market. To this end, a quantitative quasi-experimental survey will be conducted on shares traded by December 30, 2019. This is at least 90% of the time we have traded in the last 5 years. From the average Q value of the relevant economic sector, the value of each company's stock is adjusted to calculate the estimated price of the company's stock. If there is a disparity between the estimated value of the stock and the value at time t, you can forecast the stock price transition. If the computation yields a more significant result than what is now witnessed in the market, the next period's development will be positive; if it yields a lower result, its development will be negative. The results allow us to establish a significant influence of industry results on the performance of corporate Tobin's Q at the individual level. The significance level is greater than 50% in all cases, and profitability does not go below 30% in any sector, even reaching 100%. The methodology used in this study is essential to all investors tiny firms because it uses publicly available, freely available information, providing tremendous potential for safer non-business revenue.

## 1 Introduction

The financial analysis makes it possible to establish a better decision-making process based on the company's historical information to project its future behavior utilizing indicators that summarize critical aspects to be evaluated. While it is true that the information is accurate and reliable, conducting a consistent and well-structured analysis and making fair and appropriate comparisons ensures that the best possible decisions are made (Ibarra Mares, 2006). Lack of information or information that comes from untrustworthy sources is based on mere assumptions or is barely related to reality, with hasty analyses that rely more on experimental intuition than on experience or knowledge. Comparisons that are misguided or unfocused from the objectives set to ensure that bad decisions are made. "luck” and "chance” can sometimes describe an analyst. However, they will never become systematic decision-making models, and while there are significant studies aimed at studying emotions involved with the financial market prediction (Cambria et al., 2017; Minsky, 2014; Tan et al., 2019). So-called "instincts,” no matter how basic, are only helpful if they are well supported and justified by specific scientific methodologies and produce fairly positive results over time.

In this project, the *intuitive model* is widely accepted by most analysts involved in financial decision making, portfolio building, portfolio decision integration, and generally various companies, governments, and individuals. Intuitive approaches are associated with concepts such as subjective trust in people who have had some success due to unforeseen circumstances and respect for the *status quo* given by the market itself. So-called professionals, collective action, generalization due to lack of contextual knowledge, and many others correspond to the investor's instinct rather than the actual scientific basis and methodological objectivity of the acquired theoretical or empirical knowledge. Based on these assessments, the investor faces a possible scenario of returns far below the real possibilities of a well-established, systematically constructed portfolio.

According to Espitia Escuer (1986), although companies handle specific information, the financial analysis seeks to find general indicators that present information common to all organizations, such as general profitability or return on capital. Such indicators are mainly intended to favor better decision-making in financial markets, which are essential to promote economic activity through the efficient use of liquid resources. According to Rodríguez (2007), financial markets facilitate the flow of funds from surplus units to deficit units, either in the money market or in the capital market, making resources flow towards investment and financing agents, stimulating economic growth. This is why the actions of individual economic agents (companies and individuals) have effects in the macroeconomic sphere, whether in terms of expectations (Heymann, 2000), technological change (Pampillón Olmedo and Avila Lizeranzu, 2001), or financial decisions (Restoy Lozano and Molina, 2004).

From the simplest to those constructed by complex methods and tools, financial and market indicators represent methods of predicting the behavior and trends of common financial markets, especially equities (Bernstein, 1999). However, most of them show the subjectivity of decision-makers (Ibarra Mares, 2006) or at least those who depend on decision-making and are only subjective. This is mainly due to the difficulty of constructing or interpreting these methods and the lack of clear economic implications. This will force analysts to use traditional methods withstood decades of trials (Lizárraga, 1996; cited by Ibarra Mares, 2006).

At the microeconomic level, individual actors act rationally and thus play a role in maximizing profits in the market, whether financial or actual. Therefore, they need simple, practical tools to improve decision-making, results deviate from intuitive models, and ultimate predictions and recommendations., And discuss financial decisions in a more scientific depth. The goals of this type of analysis deviate from various situations that prevent them from having clear theoretical guidance (Bukovinsky, 1993; Cited by Ibarra Mares, 2006). These tools allow for a more accurate and reliable description of reality. These tools enable a more accurate and reliable description of reality. Through this type of method, a better decision-making system is based on proven traditional indicators (Lizárraga, 1996; cited by Ibarra Mares, 2006) of dividends, at least more controversial as to why these decisions are made.

Although the capacity and scope of equity forecasting methods have improved, they have a complete structure that provides relevant data with completeness, immediacy, and dissemination of information that investors need to evaluate based on multiple sources. Nothing has been successful in building a high level of trust. In the end, even with methodological advances, stock purchase and sale decisions end up being more the result of the investor's intuition and subjective perception, typically based on the compilation of information in the specialized press, the investor's feelings about the market and its movements, empirical knowledge based on experience and the occasional technical detail. However, we have developed a systematized dynamic and accurate trending approach that allows for better short, medium, and long-term decision making, adapts to future stock market behavior, and outlines and provides dependable guidance. The future and operations will be influenced by the unstable, non-seasonal, and fluctuating nature of the markets themselves and the diversity and non-uniformity of the internal elements of each country in which these markets operate. It is purely economic and financial, culturally, socially, politically, and regulatory (Tuarob et al., 2021).

The method of measuring corporate performance based on the most common indexes, such as Tobin's Q, ROE, and ROA, is used to examine the process of corporate value creation (Otero González et al., 2020). Therefore, indicators such as Tobin's Q are defined as "financial indicators that reflect the value that the market attribute to an entity concerning the exchange cost of the entity” (Montoro and Navarro, 2010, p. 33) process. This is because if the result of this ratio is greater than 1, it tends to increase the company's value, and the marginal utility of new investment is positive (Giraldo-Prieto et al., 2017). As Q relates the firm's market value with the cost of optimizing its capacity to produce goods or services, then "this is contemplated as a going concern and not as the sum of individualized assets” (Espitia Escuer, 1986, p. 135). Therefore, the values of Q that are not equilibrium values will influence decisions in terms of business expansion, investment, and use of capital, among others.

The equilibrium value of Q is 1, where the market value of the investment, represented in the stock price, is equivalent to the replacement cost of capital, in which case, the return on investment is the same as the return on capital (Lin et al., 2018). If the value is greater than 1, the investment generates an additional income to the return on assets from the stock market valuation and will encourage investment and expansion decisions. Conversely, if the value is lower than 1, the replacement cost will be higher than the stock valuation, discouraging investment.

Tobin's Q ratio is widely used as a factor in financial investment opportunities. To be considered a good ratio in investment opportunities, the positive relationship between the ratio that determines it and the future operating performance of a company must be observed. It is evident that according to the relationship between the q-ratio and future operating performance for publicly traded companies, companies with higher q-ratios experience superior long-term operating performance.

According to Giraldo-Prieto et al. (2017), a company can maintain a Q more significant than 1 in the medium and long term if it invests in intangible assets, not considered in the replacement cost or if it creates barriers to entry. Therefore, Q measures "...the economic value of the rents that remunerate intangible assets, as well as their extraordinary benefits, per unit of investment in physical capital” (Espitia Escuer, 1986, p. 136). Analyzing its application to the Peruvian economy, Montoro and Navarro (2010) demonstrate the importance of Q "...where the effect of a 1 percent change in Q on investment growth is around 0.08%” (p. 34), thus demonstrating a high degree of reliability, on the part of investors, in the value of the indicator.

Based on the reliability generated by this indicator and its results, it is logical to establish a trend prediction methodology based on it, taking advantage of the methodological rigor it was constructed and the availability of related information. However, it would be ideal for developing a mechanism for determining the specific and concrete point value in the price of the shares and then verifying it with reality. This is unlikely to be possible, which is why we speak of trend prediction, which is the process of establishing a line of behavior in the final price with a high level of confidence. Not only is it possible for the decision-making process to be proactive, but it is likely and feasible. Following a series of pre-established steps, it forms a search for factors that can predict outcome trends within a logical, systematic, and well-planned process. The resulting estimates reflect intangible results, generating perceptible profitability in dividends.

In this context, this research proposal aims to formalize a trend prediction methodology based on the relationship between the sectorial Tobin's Q with the Colombian market share price, considering that it is much more likely - and expected - that the value of a company's shares aligns with the sector's trend than the opposite. To do so, first, grasp the extent of Tobin's Q as a stock analysis approach, gather Latin American sector information to utilize with these chosen firms, and then use Sector Tobin's Q. The evolution of stock prices must be experimentally determined based on intertemporal correlations. The aforementioned has a substantial impact on the Colombian stock market from an investment standpoint.

## 2 Theoretical framework

### 2.1 Financial markets

A market is a physical or non-physical space where suppliers and buyers interact to exchange products and services. In the financial markets, the commodities exchanged are called financial assets. Therefore, this market is defined as a mechanism that connects sellers and buyers of financial instruments and facilitates transactions through the system (Court Monteverde and Terradellas Undurraga, 2010). Thanks to the growth of capital, the globalization of finance, the advancement of information systems, and the investigation of major financial crises worldwide (Montoya Pérez, 2016).

Vargas Pulido and Bayardo Martínez (2013) point out that the strength of financial markets is to provide more direct and cost-effective financing options that can share and diversify risks. The project is a higher level of innovation and profitability (López-Rodríguez et al., 2019). In this sense, the capital market is a compendium of solutions for financing and investment that facilitate business development through the value and efficiency of organizations (Xu and Gong, 2017).

Salman (2019) mentions that international financial markets are generally composed of information that establishes prices of financial assets, which facilitate the materialization of prices and products in the capital markets, which benefit all market agents. That is why the financial market allows diversifying risk and sharing it with new investors, who can also diversify it through the market itself (Siri and Serur, 2018). To this end, the owner of the funds is responsible for diversifying his investments by forming a portfolio composed of stocks with different risks and returns (Vargas Pulido and Bayardo Martínez, 2013).

Arbeláez García and Rosso (2016) identify that an increase in investment amounts in the financial market translates into a decrease in risks for agents and lower interest rates that intermediaries can offer. Hence the portfolio selection theory establishes that the risks associated with an asset and a portfolio are equal to the risk of each security as a whole and not in isolation (Kristoufek and Vosvrda, 2013). Avoiding investment risks and making accurate predictions about the stock market is essential for investors seeking more reliable, accurate, and real-time information to improve their profit margins. Researchers in this area of knowledge understand the preponderant role of these markets in the economy (Tuarob et al., 2021).

The international financial system and capital markets need to be understood as relevant scenarios used to mitigate investment and risk through the selection of different financial assets (González Bueno and Chacón Arias, 2014). In the financial market, the volatility of stock prices is significantly altered by macroeconomic conditions generally associated with changes in exchange rates and interest rates (Kristoufek and Vosvrda, 2013).

For Salman (2019), an efficient capital market should not contain speculation and, for this reason, there should be no distrust among investors or economies in the world. Capital markets promote brokerage and regulation by offering a variety of investments and leveraging alternatives (Ruffo and Costa, 2019), and taking risks in all decisions. This situation was developed by financial and economic integration between states with both positive and negative consequences (Court Monteverde and Terradellas Undurraga, 2010).

Miyazawa et al. (2019) state that global financial integration plays an essential role in economic growth, as long as the challenges and changes in economic policies generated by the continuous integrations of capital markets and their respective interactions are accepted (López-Rodríguez et al., 2019). For López Herrera et al. (2017), there should be a more significant correlation and depth between the number of investors at the international level and public and private companies, developing and standardizing in a better way the market as a whole (López-Rodríguez et al., 2020). Financial and stock markets must increase this interaction to increase investment options and favor any industrial or business sector (Malo de Molina, 2017).

There are always two conflicting positions in capital markets and any trade or financial transaction (Rincón, 2018). As Raposo and Lehmann (2019) stated, market agents, are in a (financially) deficit position. Furthermore, in another over-the-top position, integrating with players in various international markets can create dependencies and staggered or infectious effects that adversely affect all capital markets and market players. Xu and Gong (2017) also relate that it is impossible to guarantee prices and complete security in a context of integration of capital markets and financial markets due to the asymmetry of information and the vulnerability of markets to political or natural decisions, among others.

### 2.2 Solidarity finance

Undoubtedly, the capitalist financial system fulfills a primary intermediation function, channeling savings and investment with an individual profit margin. This market has non-flexible access barriers that prevent ordinary people from accessing it, especially those in the informal economy. In this context, from the social concern caused by the inequities of the financing process of the economy (Martin, 2021), Solidarity Finance emerges as an instrument to correct this situation. Through the formalization of an equitable and participatory financial system that generates socio-economic returns and a positive environmental impact, framed within the concepts and objectives of Sustainable Development.

Given that the social purpose of finance is to transform the surplus income of the population into the expansion of capacity, thereby generating new income for this population, its primary purpose is the participation of various people. It is a means of social-democratic mediation. Actors want to intervene, establish economic relations of production for various production units in the region, and lead to sustainable and sustainable human development. Such developments also mean providing society with alternatives to ensure stability in rocky areas as the world is experiencing the COVID pandemic. Different governments have decreed measures limiting productive capacity in the real sector, forcing companies to seek innovative alternatives to guarantee income and safeguard their stability. In this context of constraints on the normal functioning of society, sustainability depends on the survival of organizations and the jobs they offer.

This strategy is fundamental to maintain and expand the competitiveness of organizations or population groups through the investments it makes in the financial market in cycles in which the growth of its production does not generate perceptible returns, determining benefits for the organization in particular and society in general. For example, Tahmasebi and Askaribezayeh (2021) show that access to good financial schemes even increases individuals' level of social interactions.

To develop consistent criteria of sustainability and equity in the solidarity financial sector, socio-economic dynamics must be understood. The ideas of benefit and usefulness are also validated and affirmed in terms of reciprocal benefit. This solidarity relies on the social capital of contributions built into the financial base to interfere in the market at the community level, break the chain of product outsourcing and production, and generate other profit opportunities. Reinvestment, in other words, will be a significant factor in economic organizations.

These new perspectives on solidarity-based financing may make it possible to use techniques like stock price net asset ratios to calculate investment costs and social impact (social balance) costs for territories and their production. The foundation also assesses the profitability of this sector, the social benefits generated around it, and the investment and sustainability of the relevant socio-economic groups.

### 2.3 Financial theory

Financial theory has its origin in economics (Azofra Palenzuela and Fernández Álvarez, 1992), emphasizing essential aspects of investment, such as risk and uncertainty. It should be emphasized that the long-term resource allocation process and the determination of cost of capital and market value of a company are concepts such as resource optimization and, therefore, microeconomics (López-Rodríguez et al., 2019). Opportunity cost, frontier analysis (Flórez Ríos, 2008) and maximization of profits.

The first financial theoretical approach is empirical and oriented towards profit through production costs. Resources would be generated by the organization itself, without a significant contribution in financial market theories (Jensen and Smith, 2000). The traditional approach focuses on the demand for external funds and the investment and expenditure decisions that justify that financing. With the evolution of financial markets, the modern approach attempts to solve the problem of the optimal allocation of resources among alternative uses, framed in changing markets and in permanent innovation, in a clear link with microeconomics (Azofra Palenzuela and Fernández Álvarez, 1992). The risk assessment approach assumes that risk and uncertainty are the critical elements in globalized, integrated, and interdependent markets, subject to permanent external threats that determine their behavior (Flórez Ríos, 2008).

One of the most significant contributions to the modern approach is the Efficient Market Hypothesis, formulated by Fama (1970). This concept postulates that, for a market to be efficient, it must be highly competitive in terms of agents, information, and transaction costs, and due to this circumstance, the market price of financial assets adjusts quickly and accurately to the intrinsic price, which reflects all available information (Duarte and Pérez-Iñigo, 2013). Therefore, equilibrium occurs when the asset's market price and intrinsic price are equal.

The level of market efficiency is mainly associated with the level of information available to agents (Campbell et al., 1997). On the one hand, weak efficiency is when the price of assets represents historical information, thus being associated with technical analysis. When historical and public information is available, we speak of semi-strong efficiency associated with fundamental analysis. Finally, the two types of analysis would be surpassed by strong efficiency. The investor has at his disposal all possible information: historical, public, and privileged (private) (Uribe Gil and Ulloa Villegas, 2011).

Framed in the Efficient Market Hypothesis is the analysis of Tobin's Q since the calculation of this ratio arises from the quotient between the book price of the asset (intrinsic) and the market price (Tobin and Brainard, 1976). The equilibrium is at value 1, when the intrinsic price corresponds to the market price, showing efficiency in the sense of (Fama 1970).

Furthermore, Grossman & Stiglitz (1980) show the paradox that strong efficiency is not possible due to the high cost of privileged information and the lack of incentives to obtain or disclose information when it becomes available. It is confirmed that Q of is a related index of the semi. -Efficient market for using historical and public information (Uribe Gil and Ulloa Villegas, 2011). Of course, it also has a place in weak efficiency markets.

Nowadays, more than in other times, there is an urgent need to design theories, methods, or techniques to improve the predictive capacity of the stock market, taking into account the increasingly close and robust interconnection between the behavior of these markets and the "health” of national economies. The risks and uncertainties are inherent in financial totals to stabilize economic and economic policies aimed at providing economic and social benefits, in addition to the monetary gains associated with a successful decision-making process in this market. It is critical to developing inferences that lessen society's uncertainty (Tuarob et al., 2021).

It is important to emphasize how his predictions from non-traditional auto-learning can be part of his composition when it comes to volatility issues. Therefore, studies such as (Tan et al., 2019) highlight how nonlinear methods have demonstrated their effectiveness concerning elements such as volatility, price prediction, and stock valuation associated with market behavior. Artificial neural networks, decision trees, deep neural networks, gradient-boosted trees, and random forests stand out (Krauss et al., 2017).

The above is associated with the dynamics concerning market sentiment, which is visualized as the set of combined attitudes that investors may develop towards a specific market (Tungjitnob et al., 2021). In this regard, Kearney and Liu (2014) state that sentiment has little predictive power for future stock market returns in the short term.

### 2.4 Technical analysis

Financial decisions test all the knowledge and intuition of an investor. The fundamental goal of making an investment decision is always to profit. In most cases, the higher and faster, the better, as the profits you get are proportional to the investor's risk. Pring (2014) says that "the art of technical analysis, which in itself is an art, is to identify trend changes at an early stage and maintain an investment posture until they move beyond the evidence and identify that the trend has turned."

"There are three conventional approaches to stock price forecasting: Technical Analysis, Traditional Time Series Forecasting, and Machine Learning Method” (Selvamuthu et al., 2019). Contrary to the efficient market hypothesis that the price of a stock is a faithful reflection of the context and available information, Technical Analysis implies that these prices result from a trend with temporal modifications where its orientation changes (Rosillo et al., 2013).

Simply put, technical analysis is based on two fundamental assumptions. On the one hand, price fluctuations constitute behavior in a recognizable pattern when the signal to look for is clear, and on the other hand, technology. Physical, physical, and financial efforts included the implementation of this methodology. However, the data collected must be wide enough to identify the pattern, and they must be persistent enough to be considered as such. In the former case, trends are investigated based on statistical methods such as moving averages, and in the latter case, milestones in which these trends change direction are sought (Menkhoff and Taylor, 2007).

The plethora of quantitative and qualitative financial information available, especially in electronic media, has generated a broad interest for investors and researchers in establishing methodologies that combine the information to generate reliable predictions. However, the most incredible difficulty of Technical Analysis lies in the fact that the signals to be taken into account are not the same for all markets and all companies, the trends to be identified do not constitute a standard, ergo, the knowledge required transcends the mathematical and statistical concepts of inferential analysis, going through the understanding of a highly complex market such as the financial one, where data do not have a linear or seasonal behavior (Atkins et al., 2018a).

Technical Analysis is based on the review of historical stock prices and trading volumes, supported by graphic tools, to predict the markets' future behavior (Castro Alfaro and Anturi Santos, 2015), seeking to help the investor buy or buy or sell decisions. Therefore, technical analysis constitutes a fundamental methodology for stock market decision-making. Edwards and Magee (2011) define technical analysis as science related to recording the actual history of transactions (price changes, transaction volumes, etc.), primarily in the form of graphs. Keynes & Hicks (1936), in a complete chapter of his work, gives concepts that help to understand this phenomenon.

By trying to know the trends and changes in stock prices, Technical Analysis somehow reflects the behavior of investors, including their fears, instincts, and impulses, so that "...Technical Analysis is applied social psychology” (Elder, 2017, p. 80). For this reason, it is often chosen over other types of analysis when it comes to making predictions. In addition, it is easily supported by trend charts that reveal inflection points or the direction of movement (Murphy, 2016).

In this context, to find and clarify tangibly exploitable patterns by investors in estimating stock market behavior, Technical Analysis makes predictions of future stock prices based on quantitative analysis of time series, such as trading traffic and opening and closing stock prices (Tuarob et al., 2021).

The ability to anticipate and make well-founded predictions is essential to establish successful investment projects that combine accurate forecasts with taking advantage of the financial, stock, or aggregate markets opportunities. Many investors use different models based on historical analysis of statistical data. The so-called technical analysis or trend graphic techniques states that stock prices faithfully reflect the available information and cannot go against past market trends without broadening the risk acceptance base (Wan and Yang, 2019).

### 2.5 Fundamental analysis

The almost ironic result of a decision based on technical analysis is that the search for a stock price reversal involves a "bet” on when and how the pattern will be implemented, resulting in market instability. Many combinations of these bets will eventually lead to "self-actualization prophecies” that speculate that a situation will eventually occur. Many people determine the movement of markets and companies and their conditions. Faced with this situation, many investors have lost confidence in this type of analysis and turned to other methods, such as fundamental analysis, where stock prices are closer to the fundamentals of companies and markets. As a result, when many analysts and investors follow this method, the stock price is much in line with the company's book value (Schmitt and Westerhoff, 2014).

Studying market signals, a fundamental pillar of Technical Analysis has proven to be somewhat fallible compared to Fundamental Analysis. These signals are fundamentally linked to subjective conditions such as intuition, feeling, and practical experience. Fundamental Analysis assumes that stocks have a fair value established by accounting fundamentals and company valuation methodologies, then any discernible alteration in this fair value is an almost unambiguous indication of decisions to be made as stocks are more likely to converge to this fair value than to move away from it (Bartram and Grinblatt, 2018).

Fundamental Analysis seeks to establish the stock valuation of a company based on its economic structure, using as much information as possible and assuming that "Fundamentals are based on the fact that the value of a share is the discounted flow of the company's future profits” (Castro Alfaro and Anturi Santos, 2015, p. 8). Penman (1992) highlights the role of accounting information in this valuation methodology. Giner Inchausti et al. (2002) add that although financial statements are not the only relevant element in company valuation, market prices tend to converge rapidly with book value.

The origin of this methodology is the purchase of stocks at a low price concerning *intrinsic value*, that is, the concept of value investing that includes the components that make up fundamental analysis (Holloway et al., 2013) and was formulated from Columbia University by Graham & Dodd (1996) in their book "Security Analysis."

To efficiently predict the value of an asset, fundamental analysis integrates asset-specific qualitative data with the analysis of relevant macroeconomic variables. The former is obtained directly from profitability reports, dividend rates, financial statements, and general information on various companies. The latter is derived from national indicators published by the National Statistics Bureau, analysis by professional media outlets, and other sources of reliable macroeconomic information (Atkins et al., 2018). According to Sánchez Fernández de Valderrama (1991), Fundamental Analysis is essential for corporate investors. Accounting information is fundamental, different from those who seek short-term returns and base their decisions on technical analysis.

The formation of market prices from relevant information relates Fundamental Analysis to the Efficient Market Hypothesis (Fama, 1970). Although according to Giner Inchausti et al. (2002), fundamental analysis was somewhat left aside from the 1960s onwards, in favor of the Efficient Market Hypothesis, with models such as the Capital Asset Pricing Model (Sharpe, 1964) or the later Arbitrage Pricing Theory (Roll and Ross, 1980). However, the same authors point out a reconciliation between the two concepts through the EBO model and an important field of action for fundamental analysis in determining abnormal future results, the intrinsic value from the cost of capital, and the prediction of the result and net income.

In his book "Handbook of Fundamental Analysis,” Scherk (2014) sets out two ways in which the use of fundamental analysis can be carried out. The first is the "top-down” method, which studies the macroeconomic aspects that affect the company. This methodology is related to the Arbitrage Pricing Theory. The second method presented is Bottom-Up Analysis, which seeks to analyze values that refer to a given company and its behavior in the stock market. The method is similar to the previous one but assumes the macroeconomic environment as a given and is more oriented towards the business environment (p. 99) and would be more closely linked to methodologies such as the Capital Asset Pricing Model.

### 2.6 Tobin's Q and its applicability

The Q ratio is based on the technical analysis from the observation of market prices. However, it is linked to the fundamental analysis due to the relationship with the asset's intrinsic value. In this sense, this ratio responds to the conditions of the Efficient Market Hypothesis. Wernerfelt and Montgomery (1988) use Tobin's q to measure firm performance to estimate the relative importance of industry, focus, and shared effects. Salinger (1984) uses Tobin's q ratio to measure monopoly power and examine the relationship between market structure and profitability. Lustgarten and Thomadakis (1987) relate Tobin's q to the structural characteristics of firms and find that the relationship depends on market conditions. Blose and Shieh (1997) find a significant positive relationship between Tobin's q and the magnitude of the stock market reaction to capital investment announcements. Doukas (1995) and Lang and Litzenberger (1989) use the q-ratio to test the cash flow signaling and free cash flow or overinvestment explanations of the impact of dividend announcements on stock prices.

According to Bharadwaj et al. (1999), Tobin's q serves to examine the association between IT investments^1^ and strong q values after controlling for a variety of industry factors and firm-specific variables. Kim and Lyn (1986) use the ratio to explain the positive relationship between the excess market value of multinational corporations and the degree of international involvement as measured by the percentage of foreign sales. According to Peters and Taylor (2017), Tobin's Q is beneficial to explain investment in intangibles, which, traditionally, has adjusted more slowly to the change in investment opportunities than investment in physical assets. Finally, the Q ratio has been used to measure the impact of Corporate Social Responsibility on firm value, both from individual Q value (Luo and Bhattacharya, 2006) and sectoral value (Jo and Harjoto, 2011).

Regarding the theoretical origin of the Q concept, some proposals place it between neoclassical theory (Ahijado Quintillán, 1982; Alonso Borrego and Bentolila Chocrón, 1992; Hayashi, 1982) and Keynesian theory (Espitia Escuer, 1986; Tobin, 1969). For Espitia Escuer (1986), the Q ratio is a concept put forward by Tobin; however, its origins are attributed to Keynes in his General Theory. Tobin (1969) begins with a typical Keynesian element. For example, on the one hand, reviewing financial accounts from individual units that refer to the business sector and the economy as a whole suggests a balance with "finance.” Assets are determined by the LM curve, where interest rates balance the money market, the sector and the "real” sector, the trend of agent consumption or savings, and investment decisions in portfolios and time deposits. Even Tobin & Brainard (1976), justify the "Q” ratio based on the duality expressed by Keynes himself (1936, p. 151) between the valuation of assets in the capital market and the cost of replacing them.

Tobin and Brainard (1976) clearly define the synthesized form of the Q concept, which is "the ratio between two valuations of the same physical asset. The numerator is the market valuation: the market price of existing assets. The other, the denominator, is the replacement or reproduction cost: the market price for new resources produced” (p. 2–3). On the other hand, in the case of Ahijado Quintillán (1982), Tobin refers to the neoclassical decision of actors (individuals or businesses) based on preferences aimed at maximizing profits or profits through optimization of resources (wages and limits (Capital productivity) as a result, the goods market and the market for factors of production, including the capital, are in equilibrium. According to Alonso Borrego and Bentolila Chocrón (1992), Q has a neoclassical origin. However, in reality, it goes beyond this investment model. Including the stock market value includes the agents' expectations regarding the future value of the profitability of the company's investments. Hayashi (1982) considers Tobin's Q equivalent to the modified neoclassical investment theory.

Ahijado Quintillán (1982) points out that "this basic microeconomic framework is fully accepted by Tobin” (p. 102). However, like Espitia Escuer (1986), he strongly relates the Q concept to Keynesian theory, basing it on the marginal efficiency of capital. Likewise, Milei (2011) addresses the investment theory, which refers to the intertemporal decision between saving and spending, where he suggests that physical investment constitutes an alternative to financial saving. Therefore, the return on investment must equal the return on saving for marginal investment.

Understand investment due to the demand for investing agents. Milei (2011) frames the concepts underpinning the Theory of Investment within 2 types of models: neoclassical models, where the maximization of intertemporal profit is subject to the adjustment costs of capital as a function of investment demand, to determine the optimal level of the capital stock, and models based on Q itself, where the optimal level of capital depends on the Q quotient. (2018) starts from a critique of the neoclassical approach and Keynes and proposes Kalecki's theory, which includes elements such as expectations, profits, and practical demand effects. In any case, Q-based models end up having a neoclassical microeconomic approach, because Milei (2011) presents the value of Q as the ratio between the marginal productivity of capital and the cost of capital, based on the fact that Tobin's "Q” is dependent on the installed capital's current and future expected profit.

## 3 Methodology

The methodological proposal with which we responded to the problem posed is quantitative. It seeks to formalize a trend prediction methodology based on the relationship between the sectoral Tobin's Q and the price of shares in the Colombian market. The inquiry is quasi-experimental since it contrasts a cause-and-effect relationship, in which the circumstances of its implementation do not allow, a priori, to establish the minimum controls for its evaluation (León García & Montero García-Celay, 2006).

Initially, in a first scenario, in order to forecast the market price of a share, the main formula of Q, which is the quotient between the price of the evaluated share (Market Price per-Share) on an observed day with the book value (Book Value per-Share) of the same, is cleared. The original formula of the indicator is:

Eq. (1) Price Book Value Ratio

(1) $$\text{Price ​to ​book ​value ​ratio ​ ​}=\frac{\text{Market ​Price ​per}-\text{Share}}{\text{Book ​Value ​per}-\text{Share}}$$

Fuente: Park & Lee (2003).

Based on this, some logical adjustments need to be made to the indicators to achieve the general goal of formalizing an easily accessible generic indicator. Since the Q value calculated by the formula is the value of each company, it is changed to the average Q of the industry to which the organization to be analyzed belongs, and it is a true trend index corresponding to the behavior of the industry that can be generalized from a deductive point of view. Is generated. Under the assumption that this is the best tool to explain the established trends:

Eq. (2) Market Price per-Share Projected

(2) Market ​price ​per−share ​projected ​ ​= ​Price ​to ​book ​value ​ratio ​sectorial∗Book ​value ​per ​share

Fuente: Own elaboration.

This sector Q is the arithmetic mean of the price to book value ratio of Latin American companies in the same sector that listed their shares on the stock market during the five years prior to the period over which the projection is to be established (2019). The Latin American market was chosen because, despite its clear and marked differences, the behavior, growth traces, and response to economic and financial cycles are pretty similar, especially in the value trends of the share packages.

This average value is multiplied by the company's book value analyzed, resulting in a projected market value (Eq. (2)) that goes beyond the mere speculation of the individual share of the company observed. In order to determine a much broader trend behavior than the static analysis of the data, the variation obtained from the Market Price per-Share Projected between period t and period t+1 seeks to determine the trend of the stock price for period t+2. Subsequently, the variation obtained between period t+1 and t+2 is compared to establish the trend of the share value in period t+3, and so on until period t + n, thus applying backtesting that allows conclusions to be drawn within the research.

It is essential to clarify that the model results in a trend response. That is, it is not about establishing, projecting, predicting, or forecasting the exact or even close punctual value that the share price will have in the near future, that kind of conditions are illusory and improbable to materialize in reality and, if they were achievable, would result in such high costs that in the end would not represent any profit for whoever applies them, this methodology will result in an easily identifiable trend response. The price of the analyzed stock will have an upward or downward behavior in the study period. In the end, rigorous feedback of the trend will show if the methodology generated returns or not. Note that the critical point of the exercise is that it can provide some certainty about the results, which facilitates decisions that investors can make efficiently and effectively without resorting to the *nose of* an expert. These decisions are as crucial as answering. The question "Should I invest in this stock?"

If the Percentage Variation of the Market Price per-Share Projected result is more significant than zero (0), the decision recommended by the model described is "Invest.” On the other hand, if the resulting value is less than zero (0), similarly, the recommendation is "Do Not Invest."

Similarly, it should be noted that this model is designed to *recommend* long-term packages of stocks within a sector rather than a single stock. It aims to reduce seemingly uncertainties and ultimately ensure dividends as a package. Payments over time are achieved with more straightforward, less risky aggregates such as time deposits and government bonds.

The survey subjects consist of Colombian companies in the other commodities, credit institutions, electricity, gas and water sectors listed on the Colombia Stock Exchange for at least 90% of the period through December 31, 2019. increase. See Table 1 for the highest market capitalization in the last 5 years. The information was collected through the Economática software, a system for investment analysis that offers its users information on the capital markets of Latin America and the United States (Economática, 2019). This tool is facilitated by the financial laboratory of the Financial Administration program of the Corporación Universitaria Minuto de Dios.

**Table 1:** Companies participating in the development of the methodological strategy.

Name	Class	Country HQ	Type of Asset	Active/Canceled	Code	Economática Sector	Presence June 30, 2019
Banco Bogotá	Ord	CO	Stock	Active	Bogotá	Non-stock financial and credit intermediation institutions	98
Banco Davivienda	Pref	CO	Stock	Active	PFDAVVNDA	Non-stock financial and credit intermediation institutions	100
Bancolombia	Pref	CO	Stock	Active	PFBCOLOM	Non-stock financial and credit intermediation institutions	100
Corporación Financiera Colombiana S.A.	Ord	CO	Stock	Active	CORFICOLCF	Non-stock financial and credit intermediation institutions	92
Éxito	Ord	CO	Stock	Active	EXITO	Miscellaneous merchandise store	100
Celsia S.A Esp	Ord	CO	Stock	Active	Celsia	Electricity, gas and water companies	100
Grupo de Energía de Bogotá	Ord	CO	Stock	Active	GEB	Electricity, gas and water companies	99,8
Interconexión Eléctrica S.A. Esp	Ord	CO	Stock	Active	ISA	Non-stock financial and credit intermediation institutions	99,8

Source: Own elaboration from Economática (n.d.).

The backtesting, summarized in Results (Section 3. Tables 2, 3, 4, 5, 6, and 7), refers to verifying the certainty of the results of the model's recommendations. It is a feedback index that indicates whether or not said recommendations were the correct ones to generate returns. However, the individual results of the observations are not as relevant as the accumulated one, detailed in the Monetary Profitability Simulation, since it integrates and synthesizes the dividends of the process seen as a structure in a synergic way.

**Table 2:** Exito S.A.

**Sector: Miscellaneous Merchandise Store** **The only company in the sector**			
Percentage Variation of Market Price per-Share Projected (t - t+1)	Percentage change in Market Price per-Share (t+1 - t+2)	Model Backtesting	Simulation of Monetary Profitability (4,000,000 COP)
31/03/2015–30/06/2015: -0,4545%	30/06/2015–30/09/2015: -0,4127%	1 - Success. Disposition: Do not invest	4,000,000 COP
30/06/2015–30/09/2015: -6,4672%	30/09/2015–31/12/2015: 2,9692%	0 - Failure. Disposition: Do Not Invest	4,000,000 COP
30/09/2015–31/12/2015: 3,789%	31/12/2015–31/03/2016: 17,4624%	1 - Success. Disposition: Invest	4,698,496 COP
31/12/2015–31/03/2016: 4,7625%	31/03/2016–30/06/2016: -8,4575%	0 - Failure. Disposition: Invest	4,301,119 COP
31/03/2016–30/06/2016: 1,9650%	30/06/2016–30/09/2016: 4,7855%	1 - Success. Disposition: Invest	4,506,950 COP
30/06/2016–30/09/2016: 16,5154%	30/09/2016–31/12/2016: 2,5357 %	1 - Success. Disposition: Invest	4,621,231 COP
30/09/2016–31/12/2016: -15,3468%	31/12/2016–31/03/2017: 3,9821%	0 - Failure. Disposition: Do Not Invest	4,621,231 COP
31/12/2016–31/03/2017: -11,2346%	31/03/2017–30/06/2017: 0,2101%	0 - Failure. Disposition: Do Not Invest	4,621,231 COP
31/03/2017–30/06/2017: -0,6458%	30/06/2017–30/09/2017: 0,8619%	0 - Failure. Disposition: Do Not Invest	4,621,231 COP
30/06/2017–30/09/2017: 32,4498%	30/09/2017–31/12/2017: 7,1993%	1 - Success. Disposition: Invest	4,953,929 COP
30/09/2017–31/12/2017: -21,7716%	31/12/2017–31/03/2018: -0,4090%	1 - Success. Disposition: Do not invest	4,953,929 COP
31/12/2017–31/03/2018: -1,3761%	31/03/2018–30/06/2018: -1,3212%	1 - Success. Disposition: Do not invest	4,953,929 COP
31/03/2018–30/06/2018: -6,6378%	30/06/2018–30/09/2018: -9,6605%	1 - Success. Disposition: Do not invest	4,953,929 COP
30/06/2018–30/09/2018: -9,4806%	30/09/2018–31/12/2018: -14,5934%	1 - Success. Disposition: Do not invest	4,953,929 COP
30/09/2018–31/12/2018: 31,3354%	31/12/2018–31/03/2019: 35,8927%	1 - Success. Disposition: Invest	6,732,029 COP
31/12/2018–31/03/2019: -11,8398%	31/03/2019–30/06/2019: -13,4130%	1 - Success. Disposition: Do not invest	6,732,029 COP
31/03/2019–30/06/2019: -2,1627%	30/06/2019–30/09/2019: 22,5719%	0 - Failure. Disposition: Do Not Invest	6,732,029 COP
30/06/2019–30/09/2019: 13,9807%	30/09/2019–31/12/2019: -20,8755%	0 - Failure. Disposition: Invest	5,326,686 COP

Source: Own elaboration from Economatica (n.d.).

Model certainty: 61.11%.

Final Profitability: 33.1672%.

**Table 3:** Banco Bogotá S.A.

**Brokerage Institutions Sector** **Return on market capitalization for the last 5 years (43.62%, 114%, 110%)**			
Percentage change in projected market price per-share (tn:tn+1)	Market price per-share percentage change (tn+1:tn+2)	Model Backtesting	Simulation of Monetary Profitability (1,000,000 COP)
31/03/2015–30/06/2015: 0,9416%	30/06/2015–30/09/2015: -0,9449%	0 - Failure. Disposition: Invest	990,551 COP
30/06/2015–30/09/2015: -10,2143%	30/09/2015–31/12/2015: 1,9842%	0 - Failure. Disposition: Do Not Invest	990,551 COP
30/09/2015–31/12/2015: 16,5243%	31/12/2015–31/03/2016: 3,5355%	1 - Success. Disposition: Invest	1,025,552 COP
31/12/2015–31/03/2016: 4,4982%	31/03/2016–30/06/2016: -2,4548%	0 - Failure. Disposition: Invest	1,000,377 COP
31/03/2016–30/06/2016: 19,0917%	30/06/2016–30/09/2016: 8,2740%	1 - Success. Disposition: Invest	1,083,148 COP
30/06/2016–30/09/2016: 5,9443%	30/09/2016–31/12/2016: -2,6430%	0 - Failure. Disposition: Invest	1,054,521 COP
30/09/2016–31/12/2016: 0,5332%	31/12/2016–31/03/2017: -0,3301%	0 - Failure. Disposition: Invest	1,051,040 COP
31/12/2016–31/03/2017: 2,0581%	31/03/2017–30/06/2017: 7,6660%	1 - Success. Disposition: Invest	1,131,612, COP
31/03/2017–30/06/2017: 2,8167%	30/06/2017–30/09/2017: 10,7944%	1 - Success. Disposition: Invest	1,253,763 COP
30/06/2017–30/09/2017: 8,4570%	30/09/2017–31/12/2017: -1,0753%	0 - Failure. Disposition: Invest	1,240,282 COP
30/09/2017–31/12/2017: 7,0207%	31/12/2017–31/03/2018: 1,8707%	1 - Success. Disposition: Invest	1,263,484 COP
31/12/2017–31/03/2018: -5,2425%	31/03/2018–30/06/2018: 1,4452%	0 - Failure. Disposition: Do Not Invest	1,263,484 COP
31/03/2018–30/06/2018: -13,9716	30/06/2018–30/09/2018: -1,1615%	1 - Success. Disposition: Do not invest	1,263,484 COP
30/06/2018–30/09/2018: 7,1271%	30/09/2018–31/12/2018: -14,9800%	0 - Failure. Disposition: Invest	1,074,215 COP
30/09/2018–31/12/2018: 5,1261%	31/12/2018–31/03/2019: 23,8815%	1 - Success. Disposition: Invest	1,330,753 COP
31/12/2018–31/03/2019: -0,8770%	31/03/2019–30/06/2019: -3,6764%	1 - Success. Disposition: Do not invest	1,330,753 COP
31/03/2019–30/06/2019: 13,5502%	30/06/2019–30/09/2019: 25,8196%	1 - Success. Disposition: Invest	1,674,349 COP
30/06/2019–30/09/2019: -12,4939%	30/09/2019–31/12/2019: 7,0919%	0 - Failure. Disposition: Do Not Invest	1,674,349 COP

Source: Own elaboration from Economática (n.d.).

Model certainty: 50.00%.

Final Profitability: 67.4349%.

**Table 4:** Banco Davivienda S.A.

Percentage change in projected market price per-share (tn:tn+1)	Market price per-share percentage change (tn+1:tn+2)	Model Backtesting	Simulation of Monetary Profitability (1,000,000 COP)
31/03/2015–30/06/2015: -1,7389%	30/06/2015–30/09/2015: -10,3216%	1 - Success. Disposition: Do not invest	1,000,000 COP
30/06/2015–30/09/2015: -6,8458%	30/09/2015–31/12/2015: -9,0909%	1 - Success. Disposition: Do not invest	1,000,000 COP
30/09/2015–31/12/2015: 12,6829%	31/12/2015–31/03/2016: 28,1932%	1 - Success. Disposition: Do not invest	1,281,932 COP
31/12/2015–31/03/2016: 6,3690%	31/03/2016–30/06/2016: -2,1834%	0 - Failure. Disposition: Invest	1,253,943 COP
31/03/2016–30/06/2016: 4,1001%	30/06/2016–30/09/2016: 11,4008%	1 - Success. Disposition: Invest	1,396,902 COP
30/06/2016–30/09/2016: 9,9249%	30/09/2016–31/12/2016: 1,6949%	1 - Success. Disposition: Invest	1,420,579 COP
30/09/2016–31/12/2016: 3,40405%	31/12/2016–31/03/2017: 1,3206%	1 - Success. Disposition: Invest	1,439,339 COP
31/12/2016–31/03/2017: 4,1916%	31/03/2017–30/06/2017: 12,5668%	1 - Success. Disposition: Invest	1,620,219 COP
31/03/2017–30/06/2017: 2,7358%	30/06/2017–30/09/2017: 0,1837%	1 - Success. Disposition: Invest	1,623,196 COP
30/06/2017–30/09/2017: 8,8372%	30/09/2017–31/12/2017: -9,9820%	0 - Failure. Disposition: Invest	1,461,169 COP
30/09/2017–31/12/2017: 8,4641%	31/12/2017–31/03/2018: 1,9534%	1 - Success. Disposition: Invest	1,489,711 COP
31/12/2017–31/03/2018: -2,6061%	31/03/2018–30/06/2018: 22,8420%	0 - Failure. Disposition: Do Not Invest	1,489,711 COP
31/03/2018–30/06/2018: -14,7445%	30/06/2018–30/09/2018: -6,4499%	1 - Success. Disposition: Do not invest	1,489,711 COP
30/06/2018–30/09/2018: 4,3477%	30/09/2018–31/12/2018: -9,5906%	0 - Failure. Disposition: Invest	1,346,838 COP
30/09/2018–31/12/2018: 3,0288%	31/12/2018–31/03/2019: 32,4054%	1 - Success. Disposition: Invest	1,783,287 COP
31/12/2018–31/03/2019: 1,8066%	31/03/2019–30/06/2019: -7,6087%	0 - Failure. Disposition: Invest	1,647,602 COP
31/03/2019–30/06/2019: 12,0427%	30/06/2019–30/09/2019: 14,7820%	1 - Success. Disposition: Invest	1,891,150 COP
30/06/2019–30/09/2019: -13,4025%	30/09/2019–31/12/2019: 8,1882%	0 - Failure. Disposition: Do Not Invest	1,891,150 COP

Source: Own elaboration from Economática (n.d.).

Model certainty: 66.67%.

Final Profitability: 89.1150%.

**Table 5:** Bancolombia S.A.

Percentage change in projected market price per-share (tn:tn+1)	Market price per-share percentage change (tn+1:tn+2)	Model Backtesting	Simulation of Monetary Profitability (1,000,000 COP)
31/03/2015–30/06/2015: -1,6920%	30/06/2015–30/09/2015: -9,6313%	1 - Success. Disposition: I do not invest	1,000,000 COP
30/06/2015–30/09/2015: -7,4316%	30/09/2015–31/12/2015: -11,2564%	1 - Success. Disposition: I do not invest	1,000,000 COP
30/09/2015–31/12/2015: 7,8915%	31/12/2015–31/03/2016: 19,8780%	1 - Success. Disposition: Invest	1,198,780 COP
31/12/2015–31/03/2016: 3,1433%	31/03/2016–30/06/2016: -1,6721%	0 - Failure. Disposition: Invest	1,178,735 COP
31/03/2016–30/06/2016: 3,3273%	30/06/2016–30/09/2016: 10,5972%	1 - Success. Disposition: Invest	1,303,649 COP
30/06/2016–30/09/2016: 9,5100%	30/09/2016–31/12/2016: -2,2027%	0 - Failure. Disposition: Invest	1,274,934 COP
30/09/2016–31/12/2016: 3,1068%	31/12/2016–31/03/2017: 5,7922%	1 - Success. Disposition: Invest	1,348,780 COP
31/12/2016–31/03/2017: 4,2405%	31/03/2017–30/06/2017: 20,4085%	1 - Success. Disposition: Invest	1,624,046 COP
31/03/2017–30/06/2017: 3,1491%	30/06/2017–30/09/2017: 2,0069%	1 - Success. Disposition: Invest	1,656,046 COP
30/06/2017–30/09/2017: 7,9951%	30/09/2017–31/12/2017: -9,2210%	0 - Failure. Disposition: Invest	1,503,881 COP
30/09/2017–31/12/2017: 9,0801%	31/12/2017–31/03/2018: 0,1855%	1 - Success. Disposition: Invest	1,506,672 COP
31/12/2017–31/03/2018: -2,3432%	31/03/2018–30/06/2018: 20,2964%	0 - Failure. Disposition: No Investment	1,506,672 COP
31/03/2018–30/06/2018: -14,5564%	30/06/2018–30/09/2018: -10,3112%	1 - Success. Disposition: I do not invest	1,506,672 COP
30/06/2018–30/09/2018: 3,4962%	30/09/2018–31/12/2018: -1,3976%	0 - Failure. Disposition: Invest	1,485,615 COP
30/09/2018–31/12/2018: 6,0193%	31/12/2018–31/03/2019: 30,3178%	1 - Success. Disposition: I do not invest	1,936,020 COP
31/12/2018–31/03/2019: -0,0162%	31/03/2019–30/06/2019: 0,0092%	0 - Failure. Disposition: No Investment	1,936,020 COP
31/03/2019–30/06/2019: 12,1931%	30/06/2019–30/09/2019: 4,5168%	1 - Success. Disposition: Invest	2,023,466 COP
30/06/2019–30/09/2019: -11,8518%	30/09/2019–31/12/2019: 9,5169%	0 - Failure. Disposition: No Investment	2,023,466 COP

Source: Own elaboration from Economática (n.d.).

Model certainty: 61.11%.

Final Profitability: 102,347%.

**Table 6:** Celsia S.A. E.S.P.

**Electricity, gas, and water companies** **Return on market capitalization for the last 5 years (134%, 165%)**			
Percentage change in projected market price per-share (tn:tn+1)	Market price per-share percentage change (tn+1:tn+2)	Model Backtesting	Simulation of Monetary Profitability (1,000,000 COP)
31/03/2015–30/06/2015: -9,0744%	30/06/2015–30/09/2015: -15,1046%	1 - Success. Disposition: I do not invest	1,000,000 COP
30/06/2015–30/09/2015: -14,4884%	30/09/2015–31/12/2015: -20,9086%	1 - Success. Disposition: I do not invest	1,000,000 COP
30/09/2015–31/12/2015: 56,4705%	31/12/2015–31/03/2016: 40,3319%	1 - Success. Disposition: Invest	1,403,319 COP
31/12/2015–31/03/2016: 226,6724%	31/03/2016–30/06/2016: 2,1220%	1 - Success. Disposition: Invest	1,433,098 COP
31/03/2016–30/06/2016: -49,7499%	30/06/2016–30/09/2016: 2,5974%	0 - Failure. Disposition: No Investment	1,433,098 COP
30/06/2016–30/09/2016: 26,3556%	30/09/2016–31/12/2016: 1,8987%	1 - Success. Disposition: Invest	1,460,308 COP
30/09/2016–31/12/2016: 31,0140%	31/12/2016–31/03/2017: 8,8199%	1 - Success. Disposition: Invest	1,589,106 COP
31/12/2016–31/03/2017: -38,7361%	31/03/2017–30/06/2017: 5,8061%	0 - Failure. Disposition: No Investment	1,589,106 COP
31/03/2017–30/06/2017: 17,6627%	30/06/2017–30/09/2017: 2,5694%	1 - Success. Disposition: Invest	1,629,936 COP
30/06/2017–30/09/2017: -2,9683%	30/09/2017–31/12/2017: 2,3113%	0 - Failure. Disposition: No Investment	1,629,936 COP
30/09/2017–31/12/2017: -20,3104%	31/12/2017–31/03/2018: -4,4866%	1 - Success. Disposition: I do not invest	1,629,936 COP
31/12/2017–31/03/2018: 0,5741%	31/03/2018–30/06/2018: 3,8387%	1 - Success. Disposition: Invest	1,692,503 COP
31/03/2018–30/06/2018: -10,5135%	30/06/2018–30/09/2018: -5,6930%	1 - Success. Disposition: I do not invest	1,692,503 COP
30/06/2018–30/09/2018: 11,3146%	30/09/2018–31/12/2018: -6,8711%	0 - Failure. Disposition: Invest	1,576,210 COP
30/09/2018–31/12/2018: -45,5910%	31/12/2018–31/03/2019: 10,9322%	0 - Failure. Disposition: No Investment	1,576,210 COP
31/12/2018–31/03/2019: 1,3816%	31/03/2019–30/06/2019: 0,9244%	1 - Success. Disposition: Invest	1,590,780 COP
31/03/2019–30/06/2019: 5,1000%	30/06/2019–30/09/2019: 0,2203%	1 - Success. Disposition: Invest	1,594,285 COP
30/06/2019–30/09/2019: 5,4499%	30/09/2019–31/12/2019: 2,4743%	1 - Success. Disposition: Invest	1,633,732 COP

Source: Own elaboration from Economática (n.d.).

Model certainty: 72.22%.

Final Profitability: 63.37%.

**Table 7:** Electric interconnection S.A. E.S.P.

Percentage change in projected market price per-share (tn:tn+1)	Market price per-share percentage change (tn+1:tn+2)	Model Backtesting	Simulation of Monetary Profitability (1,000,000 COP)
31/03/2015–30/06/2015: -4,6458%	30/06/2015–30/09/2015: -1,8074%	1 - Success. Disposition: I do not invest	1,000,000 COP
30/06/2015–30/09/2015:-8,0485%	30/09/2015–31/12/2015: 5,2828%	0 - Failure. Disposition: No Investment	1,000,000 COP
30/09/2015–31/12/2015: 63,1939%	31/12/2015–31/03/2016: 17,2087%	1 - Success. Disposition: Invest	1,172,087 COP
31/12/2015–31/03/2016: 235,9427%	31/03/2016–30/06/2016: 3,3526%	1 - Success. Disposition: Invest	1,211,382 COP
31/03/2016–30/06/2016: -48,4361%	30/06/2016–30/09/2016: 10,7965%	0 - Failure. Disposition: No Investment	1,211,382 COP
30/06/2016–30/09/2016: 51,7350%	30/09/2016–31/12/2016: 3,8277%	1 - Success. Disposition: Invest	1,257,750 COP
30/09/2016–31/12/2016: 35,4355%	31/12/2016–31/03/2017: 16,2325%	1 - Success. Disposition: Invest	1,461,913 COP
31/12/2016–31/03/2017: -40,1268%	31/03/2017–30/06/2017: 15,0000%	0 - Failure. Disposition: No Investment	1,461,913 COP
31/03/2017–30/06/2017: 24,8831%	30/06/2017–30/09/2017: 4,0595%	1 - Success. Disposition: Invest	1,521,260 COP
30/06/2017–30/09/2017: 0,5658%	30/09/2017–31/12/2017: 5,2901%	1 - Success. Disposition: Invest	1,601,737 COP
30/09/2017–31/12/2017: -18,9737%	31/12/2017–31/03/2018: -6,1972%	1 - Success. Disposition: I do not invest	1,601,737 COP
31/12/2017–31/03/2018: -27,2073%	31/03/2018–30/06/2018: 8,8589%	0 - Failure. Disposition: No Investment	1,601,737 COP
31/03/2018–30/06/2018: -12,9590%	30/06/2018–30/09/2018: -5,9211%	1 - Success. Disposition: I do not invest	1,601,737 COP
30/06/2018–30/09/2018: 13,6145%	30/09/2018–31/12/2018: 6,7193%	1 - Success. Disposition: I do not invest	1,709,362 COP
30/09/2018–31/12/2018: -40,8563%	31/12/2018–31/03/2019: 27,7539%	0 - Failure. Disposition: No Investment	1,709,362 COP
31/12/2018–31/03/2019: 3,4287%	31/03/2019–30/06/2019: -10,6383%	0 - Failure. Disposition: Invest	1,527,515 COP
31/03/2019–30/06/2019: 8,8688%	30/06/2019–30/09/2019: 15,9525%	1 - Success. Disposition: Invest	1,771,192 COP
30/06/2019–30/09/2019: 0,7035%	30/09/2019–31/12/2019: 9,0694%	1 - Success. Disposition: Invest	1,931,829 COP

Source: Own elaboration from Economática (n.d.).

Model certainty: 66.67%.

Final Profitability: 93.18%.

## 4 Results

The following tables compare the estimated behavior of the shares studied in this project after running the model, compared to the value observed in reality in the period analyzed. As a way of checking the effectiveness of the model itself, it should be reiterated that the analysis refers to the certainty of the results in terms of trend. This means that the model projects whether a share has an upward or downward trend to recommend whether or not to buy into the share package, not a prediction of the specific amount to which the share price will reach.

After performing this Backtesting, it is clear that the proposed model allows formal recommendations for the decision-making process of potential investors in the stock market, giving an influential series of recommendations to generate perceptible dividends at the end of the period. In this case, with the shares used within the example stock package, the final profitability was above one-third (1/3) of the initial investment, despite certain punctual lags where more than "losing” was "not gained."

## 5 Discusión

The Efficient Market Hypothesis constitutes a methodology built throughout the study by the relationship between the intrinsic value of an asset (obtained from Tobin's Q ratio) and the evolution of market prices. The latter needs to adapt to intrinsic value (Duarte and Pérez-Iñigo, 2013; López-Rodríguez et al., 2020). The thesis of these authors is validated by the degree of significance of the model in predicting market price trends. The research results constitute a new argument favoring efficiency in financial markets.

This research is aligned with the approach of Salman (2019) because the influence of Latin American markets showed a significant impact on the behavior of the Colombian market. The results presented by the model in the three (3) sectors evaluated are consistent with what was proposed by Salinger (1984) and Lustgarten and Thomadakis (1987) regarding the decisive influence of the sector and markets on the value of Q at the corporate level. On the other hand, the research confirms what was established by Blose and Shieh (1997) regarding the significant positive relationship between Tobin's q ratio and the magnitude of stock market reaction. As a result, it is shown that the performance of regional markets decisively determines the performance of local markets and firm valuation.

The study results reiterate the importance of Tobin's Q in the aspects proposed by the author mentioned in the previous section. Some aspects of Tobin's q applicability were not tested because they are outside the scope of the current analysis. For example, the aspect of Wernerfelt and Montgomery (1988) that uses Tobin's q as a measure of corporate performance to estimate the relative importance of the industry. Focus and co-effects, as this study is proceeding in the opposite direction to that suggested in this study. On the other hand, a suggestion by Bharadwaj et al. (1999) Tobin's Q states that it is designed to investigate the relationship between investment in information and communications technology and a company's Q score. This is not part of the objectives proposed here, after controlling various industry factors and company-specific variables. It may be the subject of future research.

## 6 Conclusions

The model structured in this project efficiently exposes, in a formal manner, a specific strategy for trend prediction of stock prices in different sectors based on the relationship between sectoral Tobin's Q and stock prices. For the case of the Colombian companies analyzed, the results allow us to preliminarily induce that the methodology will work positively in other applications and markets.

The methodology constructed effectively responds to the need to offer a basis for investment decisions that are totally objective, free of value judgments, and closely linked to the widely accepted theoretical scaffolding. This result presents high levels of significance in its ability to predict trends. It is a handy tool for investors with high-risk aversion who do not trust intuition and prefer the security of the objective.

The methodology presents a level of certainty greater than or equal to 50 % in each feedback made to the recommended decisions. In comparison, the profitability associated with these decisions remained within the range of 33%–102 % based on the results obtained from the selected sample. If it is considered that during the analyzed period (2015–2019), the accumulated inflation in the country was 20.71% (4.45%; 5.36%; 4.07%; 2.89% 3.94%), the perceived genuine interest is between 13% and 82%. Considering the interest rate of fixed-term deposits during the same period (below 1.5% EA), the results are profitable in nominal terms and absolute terms.

The construction of such methodology responds to the efficiency of the markets because it involves the intrinsic value of the financial asset as an essential element for producing the market price trend, giving it a very high theoretical support. The model proposed by this research is far from speculative proposals, as it builds long-term trends that can be replicated over and over again based on previously observed periods, receiving a solid trend prediction basis for subsequent periods.

The study began by observing the data quantified by backtesting and demonstrated the model's power. However, there are significant limitations to the applicability of statistical and econometric tools to determine the extent of causality between markets and their level of risk. With this in mind, it is suggested to deepen the study of data developed by econometric methods such as Logit, Probit, and Arima to improve the quantification developed in this process. The methodology presented in this research is based on excellent concepts, but at the same time very simple, and can be used by anyone who wishes to gain security in their investment decision-making process, without the need for specialized training in the subject. Therefore, it becomes a powerful innovation tool for micro-businesses and small productive units, diversifying operational risk through non-operational income. This contribution is framed within the concept of solidarity finance.

The specialized literature has consistently highlighted the importance of innovation as a tool to promote the competitiveness of companies. This affirmation is supported by the fact that the application of innovation in investment portfolios makes it possible to maintain the viability of companies in contexts of uncertainty in their economic sectors, making them more competitive.

Companies often need to take innovative steps to secure cash flow through non-operating income in today's pandemic-like situation. The methodology proposed in this article allows businesses to maintain profits through non-managed financial investments and offset the decline in production and sales due to government-imposed hygiene restrictions.

In this way, finance serves its social function in two ways. One is to manage the surplus resources of society, in this case, the surplus that is not used for production, and the other is to provide an alternative to survive in uncertain situations. Innovations in investment methods that provide the right level of predictive power are essential to achieving the second path of financial, social functioning. Innovation is a powerful tool for competitiveness.

## Declarations

### Author contribution statement

All authors listed have significantly contributed to the development and the writing of this article.

### Funding statement

This research did not receive any specific grant from funding agencies in the public, commercial, or not-for-profit sectors.

### Data availability statement

Data will be made available on request.

### Declaration of interests statement

The authors declare no conflict of interest.

### Additional information

No additional information is available for this paper.

## Footnotes

1 Information technology
